# Childhood Lead Exposure After the Phaseout of Leaded Gasoline: An Ecological Study of School-Age Children in Kampala, Uganda

**DOI:** 10.1289/ehp.0901768

**Published:** 2010-03-01

**Authors:** Lauren K. Graber, Daniel Asher, Natasha Anandaraja, Richard F. Bopp, Karen Merrill, Mark R. Cullen, Samuel Luboga, Leonardo Trasande

**Affiliations:** 1 Yale University School of Medicine, New Haven, Connecticut, USA; 2 Global Health Center and; 3 Department of Pediatrics, Mount Sinai School of Medicine, New York, New York, USA; 4 Department of Earth and Environmental Sciences, Rensselaer Polytechnic Institute, Troy, New York, USA; 5 Department of Medicine, Stanford University Medical Center, Palo Alto, California, USA; 6 Makerere University School of Medicine, Kampala, Uganda; 7 Department of Preventive Medicine, Mount Sinai School of Medicine, New York, New York, USA

**Keywords:** children, dust analysis, leaded gasoline, lead poisoning, soil analysis, Uganda

## Abstract

**Background:**

Tetraethyl lead was phased out of gasoline in Uganda in 2005. Recent mitigation of an important source of lead exposure suggests examination and re-evaluation of the prevalence of childhood lead poisoning in this country. Ongoing concerns persist about exposure from the Kiteezi landfill in Kampala, the country’s capital.

**Objectives:**

We determined blood lead distributions among Kampala schoolchildren and identified risk factors for elevated blood lead levels (EBLLs; ≥ 10 μg/dL).

**Analytical approach:**

Using a stratified, cross-sectional design, we obtained blood samples, questionnaire data, and soil and dust samples from the homes and schools of 163 4- to 8-year-old children representing communities with different risks of exposure.

**Results:**

The mean blood lead level (BLL) was 7.15 μg/dL; 20.5% of the children were found to have EBLL. Multivariable analysis found participants whose families owned fewer household items, ate canned food, or used the community water supply as their primary water source to have higher BLLs and likelihood of EBLLs. Distance < 0.5 mi from the landfill was the factor most strongly associated with increments in BLL (5.51 μg/dL, *p* < 0.0001) and likelihood of EBLL (OR = 4.71, *p* = 0.0093). Dust/soil lead was not significantly predictive of BLL/EBLL.

**Conclusions:**

Lead poisoning remains highly prevalent among school-age children in Kampala. Confirmatory studies are needed, but further efforts are indicated to limit lead exposure from the landfill, whether through water contamination or through another mechanism. Although African nations are to be lauded for the removal of lead from gasoline, this study serves as a reminder that other sources of exposure to this potent neurotoxicant merit ongoing attention.

Knowledge about the threat posed by lead exposure to children’s learning and development dates at least back to 1904, when J. Lockhart Gibson documented the harm suffered by children who chewed on the painted walls and verandas of houses (Gibson 1904). Although it was believed originally that levels of 40–80 μg/dL were required to result in adverse impacts on children’s health, studies have suggested that blood lead levels (BLLs) < 10 μg/dL may be associated with decrements in childhood intellectual quotient (Canfield et al. 2003; Jusko et al. 2008; Laraque and Trasande 2005). In the United States and Europe, epidemiologic evidence has driven the successful removal of lead from gasoline and paint, resulting in average BLLs in the United States today that are one-tenth of what they were in the late 1970s (Grosse et al. 2002; Jones et al. 2009). Predictable reductions in BLL have been described in other countries as well (Thomas et al. 1999). A South African study of 5- to 11-year-old children identified 10% with elevated blood lead levels (EBLLs; ≥ 10 μg/dL), a 70% decrease 6 years after the phaseout of lead in gasoline began compared with a study of the same population before the phaseout (Mathee et al. 2006).

In much of the world, however, progress to reduce exposure to this neurotoxicant has been slower. Although countries such as Uganda have recently phased out lead from gasoline, exposure to lead in Africa is still widespread, with the greatest risk of contact coming from lead in paint, mining, lead-smelting facilities, polluted waters, contaminated foods, and residual lead from prior gasoline use. The factories and landfills that have not been properly abated of lead pose significant risks, possibly resulting in contamination of groundwater (Nriagu et al. 1996). In Uganda, where the gasoline lead phaseout was completed in 2005 [United Nations Environment Programme (UNEP) 2009], the Kiteezi landfill site in Kampala has been found to contain high levels of lead and other heavy metals (Mwiganga and Kansiime 2005). Thirty-five percent of families supplement their meals with food from gardens and farms that line the roadways in Kampala and ingest the lead in and on their food crops (Nabulo et al. 2006).

The Kiteezi landfill, located 12 km north of Kampala City, ends in a wetland through which the Kitetikka stream runs. The Kitetikka stream feeds some of the community water supply (Figure 1), and there are two cattle farms located on either side of the landfill. A study of environmental samples has found lead contamination in leachate from the landfill as high as 1 mg/L. Levels up to 0.4 mg/L lead were reported in Kitetikka streamwater downstream of the landfill, about four times higher than at upstream sites (Mwiganga and Kansiime 2005). Given that the withdrawals from Kitetikka stream downstream of the landfill constitute a community water source and that water contamination with lead can contribute significantly to BLLs in children (Edwards et al. 2009; Maas et al. 2005; Miranda et al. 2007; Troesken 2006), concern in the communities that comprise Kampala City about preventable lead exposure is not surprising.

Community concern about the landfill led to the design of a study whose purposes were to determine blood lead distributions among Kampala schoolchildren and to identify risk factors for raised BLLs, including home proximity to the landfill. Using a stratified, cross-sectional study design, we obtained BLLs, questionnaire data, and soil and dust samples from the homes and schools of 163 4- to 8-year-old children representing communities with different risks of exposure.

## Methods

### Human subjects and setting

Nine schools with varying degrees of proximity to the Kiteezi landfill were selected for this cross-sectional study. All nine schools are within 1.5 miles of the Kiteezi landfill and were selected for the study under the guidance of the Ugandan Ministry of Education and Sports. Data collection was completed during June–August 2009. The study protocol was approved by the Institutional Review Boards of Yale University School of Medicine, Mount Sinai School of Medicine, Makerere University Faculty of Medicine, and the Uganda National Council for Science and Technology.

The parents of all participants were fully informed about the purposes and limitations of the study and provided a written consent in either Luganda or English. All documents were translated and back-translated into Luganda, the primary language of the region, and were reviewed by a local focus group. Letters explaining the study were sent to all children 6 or 7 years of age (*n* = 400) at the nine Kampala area schools. Of these, 163 (40.8%) provided parental consent and participant assent and were included in the study. Parents needed to be present with their children to participate. The consent forms were read verbally in Luganda to each participant prior to the study, and time was allocated for questions. After obtaining written parental consent and verbal child assent, parents were interviewed, and blood samples were obtained from the children. Community leaders were identified to locate the children’s homes for soil collection. Information on lead poisoning was provided for each family for reference.

### Blood collection and analysis

A trained laboratory technician collected 50 μL capillary blood samples by fingertip puncture using universal precautions and standard supplies. BLLs were assessed using a portable LeadCare I CLIA-waived Blood Lead Testing System (ESA Biosciences Inc., Chelmsford, MA, USA), which has been used with high reliability in previous field studies (Counter et al. 1998). Lead levels were verified by the Yale University Laboratory in Connecticut comparing capillary lead levels with 3-mL antecubital venous samples obtained from 18 randomly selected students (*r* = 0.91). Hemoglobin levels were tested using a HemaCue hemoglobin testing device (Leo Diagnostics, Helsingborg, Sweden).

### Questionnaire instrument

We adapted a questionnaire used in a study of childhood lead exposure in the Philippines (Riddell et al. 2007). Interview questions were a part of a structured, four-part oral interview: part 1, demographic information and personal ownership; part 2, home situation, water access, and land use; part 3, food and nutrition; and part 4, the health of the child and family. Medical students at Makerere University verbally read the questionnaire to all parents of the children and verbally translated questionnaires for participants who spoke a language other Luganda or English.

Interview questions included the following topics: sex, age, presence/absence of painted walls, presence/absence of chipping paint, flooring type (dichotomized as cement/tile or ground), water source (dichotomized as community water system or bore hole/dug well), ingestion of canned foods, presence of smokers in the home, and traffic patterns near the home (dichotomized as busy or very busy versus normal, slow, or very slow). Socioeconomic status was assessed by ownership of the following items: radio, air conditioner, television, living room set, dining set, refrigerator, cell phone, VCR/DVD player, landline telephone, car, and washing machine. The number of these items owned was summed to produce a continuous scale (0–11) to represent socioeconomic status for purposes of this population, to which we refer hereafter as the “socioeconomic status scale.” Focus group members suggested that asking participants to state their income would be culturally inappropriate and would prompt suspicion. Risk of take-home occupational exposures was assessed by inquiring whether a parent/other family member was in one of the following occupations/hobbies: gas station attendant, home renovation, battery factory, automobile worker, welding, pottery, stained glass manufacture, or use of firearms. Parents were asked to describe any medical problems the child experienced and were asked to list any herbal remedies/medications regularly taken by the child. Report of clay-containing remedies/medications was examined as a possible risk factor for lead exposure, as was report of malarial illness in the child, in light of a study in Nigeria that identified high prevalence of EBLL among children with malaria (Nriagu et al. 2008).

### Environmental specimen collection

Cylindrical plastic tubes and scoops were used to collect samples of approximately 10 g from the top 1 cm of soil. Dust samples (0.1–15 g) were collected from the floors, regardless of flooring integrity, by sweeping with a foam brush over a 2-ft × 2-ft square (4 ft^2^) area into a plastic dust pan. One set of dust and soil samples was obtained from each school and from each home where a study participant lived. The samples were stored in plastic bags at ambient temperature prior to analysis at Rensselaer Polytechnic Institute. All soil samples were analyzed using a XEPOS X-ray fluorescence spectrometer (Spectro, Kleve, Germany) to measure concentrations of lead. Quantification was accomplished using the TurboQuant Powder software from the same manufacturer. Analyses of soil and sediment Standard Reference Materials gave lead concentrations within a few percent of certified values with sample sizes as small as 1.2 g [Supplemental Material, Figures S-1 and S-2 (doi:10.1289/ehp.0901768)]. Smaller dust samples were analyzed after mixing with powdered silica to a total mass of 1.5 g. Dilution of dust samples by up to 15 times (0.1–1.5 g) with powdered silica or alumina was shown to have no significant effect on precision of analysis (Supplemental Material, Figures S-3 and S-4). Twenty soil samples and seven dust samples were analyzed in duplicate or triplicate. The average relative standard deviation was 3.4% for replicate soil analyses and 1.7% for dust. Details of the quality control analyses and the dilution technique applied to the lighter dust samples are reported in the Supplemental Material.

Geographic information systems (GIS) mapping of the schools and homes was employed to assess proximity to the Kiteezi landfill. Horizontal locations (latitude and longitude) were determined using a Garmin (Olathe, KS USA) Geko 201 12 channel geographic positioning system. The accuracy indicated on the instrument ranged from 6 to 20 feet. Distances were determined by entering the coordinates into Google Earth (earth.google.com/) and using the ruler tool to measure the distance from the school locations provided to the nearest landfill boundary.

### Statistical analysis

We performed descriptive, univariate, and multivariable regression analysis with four different dependent variables [blood lead, presence of blood lead ≥ 10 μg/dL (EBLL), hemoglobin and hemoglobin ≤ 11.5 μg/dL, which represents a generally accepted cutoff for anemia among 6- to 7-year olds] (Robertson and Shilkofski 2005). For blood lead and presence of elevated blood lead (≥ 10 μg/dL, the current Centers for Disease Control and Prevention action level), we examined the following independent variables: sex, age, presence/absence of painted walls, presence/absence of chipping paint, water source, ingestion of canned foods, presence of smokers in the home, busy/slow traffic patterns near the home, take-home exposure risk, history of malaria, distance to the Kiteezi landfill, use of clay-containing remedies/medications, socioeconomic status, home dust lead, home soil lead, school soil lead, and school dust lead. Distance to the Kiteezi landfill was dichotomized into < 0.5 mile and ≥ 0.5 mile, whereas soil and dust lead results were dichotomized into < 40 μg/foot^2^ and ≥ 40 μg/foot^2^ (the current U.S. Environmental Protection Agency federal standard for floor dust) (Gaitens et al. 2009). Because of modest correlation (*r* = 0.10) between soil and dust samples in homes, separate analyses were performed using soil and dust samples in predicting BLLs. Soil and dust leads were examined both as continuous and dichotomized variables. Variables found to be significant at the *p* = 0.10 level for blood lead and the presence of elevated blood lead were incorporated in subsequent multivariable regressions. Univariate regression analyses were used to examine hemoglobin levels and anemia as well as BLLs and the presence of elevated blood lead. Statistical significance was defined as *p* ≤ 0.05.

## Results

Most of the children in the study were female (56.4%) and although the ages ranged from 4 to 8 years, the mean age of the children was 6.7 years. Using our socioeconomic status scale, the average family included in the study owned 4.2 items. Many families reported to have take-home exposures (58.9%), painted homes (50.9%), and chipping paint (30.7%). More than 68% of respondents used bore holes or dug wells for their drinking water. Some parents (4.2%) reported giving their children “mululuza,” an herbal plant medication used in the treatment of malaria, helminths, constipation, diabetes, and cancer. Fewer parents (3.7%) treated their children’s illnesses with clay-containing medication. Further descriptive information about the study population is included in Table 1. Of the 163 children tested, 33 (20.5%) were found to have EBLL. The BLLs ranged from 0 to 30 μg/dL, and no children required chelation therapy. A smaller percentage (5.6%) of the children had hemoglobin levels below 11.5 g/dL. Children more frequently had EBLLs south and southeast of the landfill, with a tendency for higher BLLs near roadways.

Univariate analysis showed that participants whose families owned more household items had lower BLLs (Table 2; *p* = 0.025), suggesting an inverse relationship between our socioeconomic status scale and BLLs. Homes that used bore holes or dug wells for water had, on average, 1.97 μg/dL lower BLLs (*p* = 0.03) and one-third of the chance of having EBLL (Table 2). Consumption of canned food was also associated with a mild increase in BLL (*p* = 0.043). Although distance to the Kiteezi landfill as a continuous variable was not statistically significant, distance < 0.5 miles from the Kiteezi landfill was associated with a 4.75 μg/dL increment (*p* = 0.0002) in blood lead. Children living within 0.5 miles of the landfill were 3.4 times more likely to have EBLLs [95% confidence interval (CI), 1.23–9.45] than children living farther away. Sex, age, smoking, take-home exposures, traffic patterns, presence of paint, and chipping paint were not associated with differences in BLL or EBLL. Dust/soil lead was not significantly predictive of BLL or EBLL, whether treated as a continuous or nominal variable.

Multivariable analysis (Table 3) further supported associations of community water source use with increases in BLLs. Use of bore hole/dug well as primary water source was associated with 2.23 μg/dL decrement in blood lead and one-third the likelihood of EBLL (*p* = 0.018). Ingestion of canned food was no longer significantly associated with increased blood lead, whereas a one-point increase in socioeconomic status using our scale was associated with a 0.57 μg/dL decrease in blood lead (*p* = 0.0094). Distance < 0.5 miles from the Kiteezi landfill was the factor most strongly associated with increments in BLL (5.51 μg/dL; *p* < 0.0001) and increased likelihood of EBLL [odds ratio (OR) = 4.71; *p* = 0.0093).

## Discussion

The major finding of this study is that lead poisoning remains highly prevalent among school-age children in Kampala. Although there are no comparison data predating the phaseout of lead from gasoline in Uganda, the prevalence of EBLLs in 2008 among Uganda schoolchildren (20.2%) is comparable with albeit somewhat higher than that experienced as the phaseout was ongoing in South Africa (10%). The prevalence of EBLLs among Kampala children is likely to have been on the order of 100%, as it was in South Africa before the phaseout began there (Mathee et al. 2006) and reinforces the profoundly positive impact this intervention has had across many African countries in the past decade. Residual elevations in blood lead among schoolchildren after the phaseout of lead in gasoline may be a product of bone lead stores (Landrigan and Todd 1994) accumulated during earlier periods of life that entailed exposure to leaded gasoline, just as children whose homes have been remediated for lead paint may not have as dramatic reductions in BLLs as expected (Gwiazda et al. 2005; Rust et al. 1999). Resuspension of contaminated dust dating to the era of leaded gasoline by motor vehicles is another mechanism to consider.

Although efforts were made to obtain a cross-sectional sample of participants that represent the scope of possible exposures in this population, this study did not intend to assess prevalence of EBLLs in a representative sample of Kampala schoolchildren. It is possible that our effort to examine relationships between proximity to the Kiteezi landfill and EBLLs contributed to a higher prevalence of EBLL than that which would be expected using a random sampling design. The modest recruitment rate (40.8%) also limits external validity.

We endeavored when possible to assess exposure risks using existing and preferably validated instruments. Although the socioeconomic scale was not validated, annual income does not necessarily represent economic productivity or socioeconomic status in Uganda, because farming and other nonpecuniary work activities would not be measured by this metric. It was also not feasible in the cultural context, and we therefore chose ownership of items that represent available disposable income instead. We did not identify, as one Nigerian study did (Nriagu et al. 2008), associations of malarial illness or treatment with elevations in BLLs. This finding may represent coexisting exposures that otherwise explain the exposure–biomarker relationship in the setting of self-reported malarial symptoms or antimalarial use.

Our pilot study funding did not permit testing paint in the field to assess completely for the presence of leaded paint as a route of exposure. However, in contrast to the United States, where dust lead is the strongest predictor of BLLs (Dixon et al. 2009), proximity to the Kiteezi landfill, lower socioeconomic status, consumption of canned foods, and use of a community water system were each associated with increases in BLLs in this population. Our prevalence of elevated dust lead (18.1%) was much higher than that most recently documented in a nationally representative sample of homes of U.S. children (4.0%) (Gaitens et al. 2009), and the testing of paint would have otherwise enhanced our capacity to identify exposure–biomarker relationships. Our failure to identify such a relationship in this population could be the product of more significant exposures from the landfill and water sources that overwhelm our capacity to detect dust–blood lead relationships in this sample of 163 children who may otherwise have been identified in a larger sample. The lack of a relationship between dust lead levels and BLLs may also be a function of the age of the children, because school-age children have less hand-to-mouth activity than younger ones (National Research Council 1993). We also noted heterogeneity in the characteristics of dust that may reduce or increase inhalability or ingestibility of the lead contaminant and may result in differences in BLLs given the same dust lead concentration [see Supplemental Material, Sample Preparation (doi:10.1289/ehp.0901768)].

The strong association of socioeconomic status with elevated blood lead is likely to represent other coexisting risk factors that may explain this association and is consistent with other studies. Although the relationship between canned food ingestion and BLLs did not persist in the multivariable analysis, lead could conceivably be in the solder of the cans or could be from another exposure found in the homes where canned foods are consumed. This can be confirmed only through further analysis of canned food samples and cans. Although geographic distribution suggested a propensity to higher BLLs with proximity to major roads, statistical analysis did not produce evidence of this *a priori* possible association (because of relatively recent use of leaded gasoline). The prevalence of lead metal within tires in Uganda is not known, but other studies have shown that this can contribute to increased soil lead levels near roadways (Horner 1996). Major roads also emanate from the landfill and thereby complicate disaggregation of effects relating to living near roads where old leaded dust may be resuspended by vehicles from effects relating to water contamination from the landfill. We were somewhat reassured about this concern with autocorrelative phenomena by the absence of a relationship between home/school dust lead and BLL/EBLL.

The strongest association we identified on univariate and multivariable regression analysis was proximity to the Kiteezi landfill. Concerns about waste management at the site have been voiced for many years (Mugagga 2006; Mwiganga and Kansiime 2005). Indeed, two samples we obtained just inside the landfill had 87 ppm and 51 ppm lead, respectively. A sample of water treatment facility sludge, which were settled particles and otherwise separated from landfill leacheate/runoff, had 380 ppm. Association of EBLLs with proximity to hazardous waste sites has been frequently documented in the past, especially at Superfund waste sites in the United States (Lorenzana et al. 2003). A recent study associating proximity to electronic waste sites with elevations in BLLs in China (Huo et al. 2007) raises further concerns about electronic waste use in the population we studied in Uganda. Increasingly, electronic waste is being shipped to African locations for processing and recycling (Schmidt 2006). A report by the United Nations Industrial Development Program identified informal collectors of scrap computer monitors from the landfill (Wasswa and Schluep 2008). Although the landfill is not supposed to receive hazardous waste, measures are not in place to avoid mixing of electronic waste with other solid wastes.

Lead exposure through contaminated water has a long history dating at least as far back as 1868 with the death of a New York man (Troesken 2006) and remains an issue even today in the United States (Edwards et al. 2009), where it is thought to contribute 14–20% of exposure in the United States (Maas et al. 2005). Water treatment systems can be useful in preventing leaching from lead-soldered pipes and minimizing exposure (Miranda et al. 2007). In light of reports of lead-contaminated polyvinyl chloride pipes (Al-Malack 2001; Wong et al. 1988), our findings raise concerns about contamination of the existing water supply from the Kiteezi waste site (or other sources) or from contamination from the pipes that transmit water to their collection point. Indeed, Lake Victoria is a major source of tap water for Kampala communities, and Lake Victoria water (0.43–1.23 mg/100 mL) and tap water samples (0.14 mg/100 mL) have been found to have lead concentrations well above the World Health Organization maximum limit (0.001 mg/100 mL) (Mghweno et al. 2008). Although lower levels also contribute to increases in blood lead (Lanphear et al. 1998), contamination in the range identified in Lake Victoria could explain a significant proportion of elevations in blood lead in this population alone (Pocock et al. 1983). The lack of water sample data, however, limits our ability to determine the contribution of lead in water to BLLs in children. The persistent finding of EBLLs in populations close to the landfill, even when water source was included in the regression model, is also concerning and merits further investigation to assess other possible routes of exposure. More work is needed to closely define the routes of exposure. The persistence of EBLL in Kampala mirrors that of Chennai, India, where EBLL remained prevalent > 4 years after phaseout of lead from gasoline and was associated with proximity to industrial activity and use of brass/bronze drinking vessels (Roy et al. 2009).

Ongoing efforts by African countries at the UNEP to eliminate lead from gasoline (UNEP 2009) are to be lauded, because they have greatly improved the cognitive potential of future generations of children. However, this study is a reminder that other sources of exposure to this potent neurotoxicant exist and merit continued attention, not only to removal of lead from paint, but also to other preventable hazards in the environment. As experience from the United States suggests, removal of these other factors can have equally great economic benefits as well (Gould 2009; Nevin et al. 2008).

## Figures and Tables

**Figure 1:**
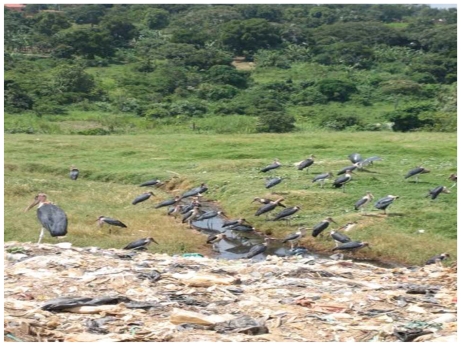
The Kiteezi landfill ends in a wetland through which the Kitetikka stream runs.

**Table 1:** Study population characteristics.

Characteristic	Value
Dependent variable	
Blood lead (capillary) (μg/dL) (*n* = 163)	7.15 ± 5.29
Blood hemoglobin (μg/dL) (*n* = 163)	12.7 ± 1.2
EBLL (≥ 10 μg/dL)	33 (20.5)
Anemia (≤ 11.5 μg/dL)	9 (5.6)

Independent variable	
Female sex	92 (56.4)
Painted home	83 (50.9)
Chipping paint	50 (30.7)
Use community water system for water source (vs. bore hole/dug well)	50 (31.3)
Consume canned food	37 (23.1)
Smokers in home	23 (14.2)
High or very high traffic	107 (63.7)
Treatment with mululuza	7 (4.2)
Treatment for malaria	28 (16.8)
Use of medication containing clay	6 (3.6)
Flooring in home	14 (8.5)
Distance to Kiteezi landfill < 0.5 mile	20 (14.2)
Age (years) (*n* = 163)	6.70 ± 0.78
Mean socioeconomic scale (0–11) (*n* = 163)	4.2 ± 2.1
Lead concentration in soil outside home (μg/foot^2^) (*n* = 115)	35.3 ± 11.9
Lead concentration in soil outside school (μg/foot^2^) (*n* = 163)	27.3 ± 8.8
Lead concentration in dust outside home (μg/foot^2^) (*n* = 94)	44.8 ± 109.6
Lead concentration in dust outside school (μg/foot^2^) (*n* = 8 schools attended by 141 participants)	28.6 ± 8.4
Risk of take-home exposures	96 (58.9)
Elevated soil lead in home	29 (25.2)
Elevated soil lead in school	17 (10.4)
Elevated dust lead in home	17 (18.1)
Elevated dust lead in school	7 (5.0)

Values are *n*(%) or mean ± SD.

**Table 2:** Results of univariate analysis of BLLs and EBLL.

Independent variable	Increment in BLL (μg/dL) compared with referent group (*p*-value)	OR for EBLL (95% CI)
Male sex (compared with female referent)	0.880 (0.298)	1.50 (0.70–3.24)
Painted home (compared with nonpainted referent)	−1.039 (0.215)	0.74 (0.34–1.59)
Chipping paint (compared with absence of chipping paint)	−0.349 (0.700)	0.80 (0.34–1.86)
Use bore hole/dug well for water source (vs. community water system)	−1.970 (0.031)*	0.33 (0.12–0.93)*
Consumption of canned food (vs. none)	2.026 (0.043)*	0.96 (0.19–4.83)
Presence of smokers in home (vs. absence)	0.985 (0.429)	1.66 (0.59–4.68)
High or very high traffic near home (vs. normal, slow, or very slow traffic)	−0.343 (0.694)	1.11 (0.27–4.63)
Take-home risk (vs. absence)	1.179 (0.165)	1.56 (0.70–3.47)
Use of mululuza medication (vs. non-use)	1.662 (0.419)	3.10 (0.66–14.59)
Treatment for malaria (vs. absence)	0.301 (0.788)	0.86 (0.30–2.48)
Treatment with clay-containing medication (vs. absence)	0.586 (0.792)	2.00 (0.35–11.42)
Presence of flooring (vs. absence)	−1.86 (0.227)	0.56 (0.16–1.96)
Distance to Kiteezi landfill < 0.5 mile	4.75 (0.0002)	3.40 (1.23–9.45)*
Age (years)	−0.677 (0.266)	0.78 (0.46–1.32)
Socioeconomic status scale	−0.464 (0.025)*	0.80 (0.65–0.99)*
Soil lead level in home	−0.054 (0.225)	1.01 (0.97–1.05)
Soil lead level in school	0.051 (0.287)	1.01 (0.97–1.06)
Dust lead level in home	0.0029 (0.520)	1.00 (0.99–1.01)
Dust lead level in school	0.019 (0.734)	1.01 (0.96–1.06)
Elevated soil lead level in home	−0.047 (0.967)	0.41 (0.05–3.51)
Elevated soil lead level in school	0.388 (0.776)	1.47 (0.27–7.99)
Elevated dust lead level in home	1.450 (0.272)	1.43 (0.40–5.10)
Elevated dust lead level in school	0.744 (0.722)	1.22 (0.22–6.85)

* *p* < 0.05.

**Table 3:** Results of multivariable analyses of blood lead and EBLL.

Independent variable	Increment in BLL (μg/dL)compared with referent group (*p*-value)	OR for EBLL (95% CI)
Use bore hole/dug well for water source (vs. community water system)	−2.23 (0.018)*	0.34 (0.11–1.05)
Socioeconomic status scale	−0.57 (0.0094)*	0.80 (0.62–1.04)
Eat canned food	0.94 (0.353)	
Distance to Kiteezi landfill < 0.5 mile	5.51 (< 0.0001)*	4.71 (1.47–15.15)*

* *p* < 0.05
